# Fixed or flexible? Orientation preference in identity and gaze processing in humans

**DOI:** 10.1371/journal.pone.0210503

**Published:** 2019-01-25

**Authors:** Valérie Goffaux

**Affiliations:** 1 Psychological Sciences Research Institute (IPSY), UC Louvain, Louvain-la-Neuve, Belgium; 2 Institute of Neuroscience (IONS), UC Louvain, Brussels, Belgium; 3 Department of Cognitive Neuroscience, Maastricht University, Maastricht, The Netherlands; Brandenburgische Technische Universitat Cottbus-Senftenberg, GERMANY

## Abstract

Vision begins with the encoding of contrast at specific orientations. Several works showed that humans identify their conspecifics best based on the horizontally-oriented information contained in the face image; this range conveys the main morphological features of the face. In contrast, the vertical structure of the eye region seems to deliver optimal cues to gaze direction. The present work investigates whether the human face processing system flexibly tunes to vertical information contained in the eye region when processing gaze direction. Alternatively, face processing may invariantly rely on the horizontal range, supporting the domain specificity of orientation tuning for faces and the gateway role of horizontal content to access any type of facial information. Participants judged the gaze direction of faces staring at a range of lateral positions. They additionally performed an identification task with upright and inverted face stimuli. Across tasks, stimuli were filtered to selectively reveal horizontal (H), vertical (V), or combined (HV) information. Most participants identified faces better based on horizontal than vertical information confirming the horizontal tuning of face identification. In contrast, they showed a vertically-tuned sensitivity to gaze direction. The logistic functions fitting the “left” and “right” response proportion as a function of gaze direction were indeed steeper when based on vertical than on horizontal information. The finding of a vertically-tuned processing of gaze direction favours the hypothesis that visual encoding of face information flexibly switches to the orientation channel carrying the cues most relevant to the task at hand. It suggests that horizontal structure, though predominant in the face stimulus, is not a mandatory gateway for efficient face processing. The present evidence may help better understand how visual signals travel the visual system to enable rich and complex representations of naturalistic stimuli such as faces.

## Introduction

The nature of the visual information being extracted when processing faces is a matter of ongoing debate (e.g., [1–5]). Vision begins with the encoding of luminance variations (i.e., contrast), at specific spatial scales and orientations (e.g., [6]). In the face image, for example, the eyebrows are defined by relatively coarse and horizontally-oriented contrast whereas eyelashes are primarily defined by fine and vertical luminance variations. Several works showed that humans identify their conspecifics best based on the horizontally-oriented information contained in the face image ([7–11]; see also [12–15] for evidence on emotional expression processing). This line of research offers a systematic and objective characterization of the visual information driving human face perception. The preferential reliance on horizontal information when identifying faces, here referred to as horizontal tuning, predicts individual differences in face recognition ability [11], drives the main behavioral and neural signatures of face-specialized processing [8, 16–18], and accounts for the recognition of both familiar and unfamiliar faces [7, 10, 11, 19]; traditional constructs in terms of e.g. featural, holistic and configural information/processing have so far failed to provide such a unified account to the processing of familiar and unfamiliar faces (e.g., [3, 20]). Beyond the theoretical implications for the field of face perception, the horizontal tuning of face identification suggests a relative preservation of orientation selectivity in high-level face processing. Disentangling whether such feature preservation reflects the privileged feedforward transmission of oriented signals from primary to high-level visual cortex, or whether it results from the feedback influence of the latter on the former [16] will impose new fundamental constraints for current theories of vision.

Despite the heuristic value of these findings for face perception and vision in general, the functional properties of orientation tuning when processing faces are poorly understood. An important unanswered question is whether it is a fixed, domain-specific characteristic, i.e. face processing invariably attunes to the horizontal range, or whether it is a flexible, task-dependent property of human face perception with different orientations being sampled according to their task relevance.

**Fig 1:**
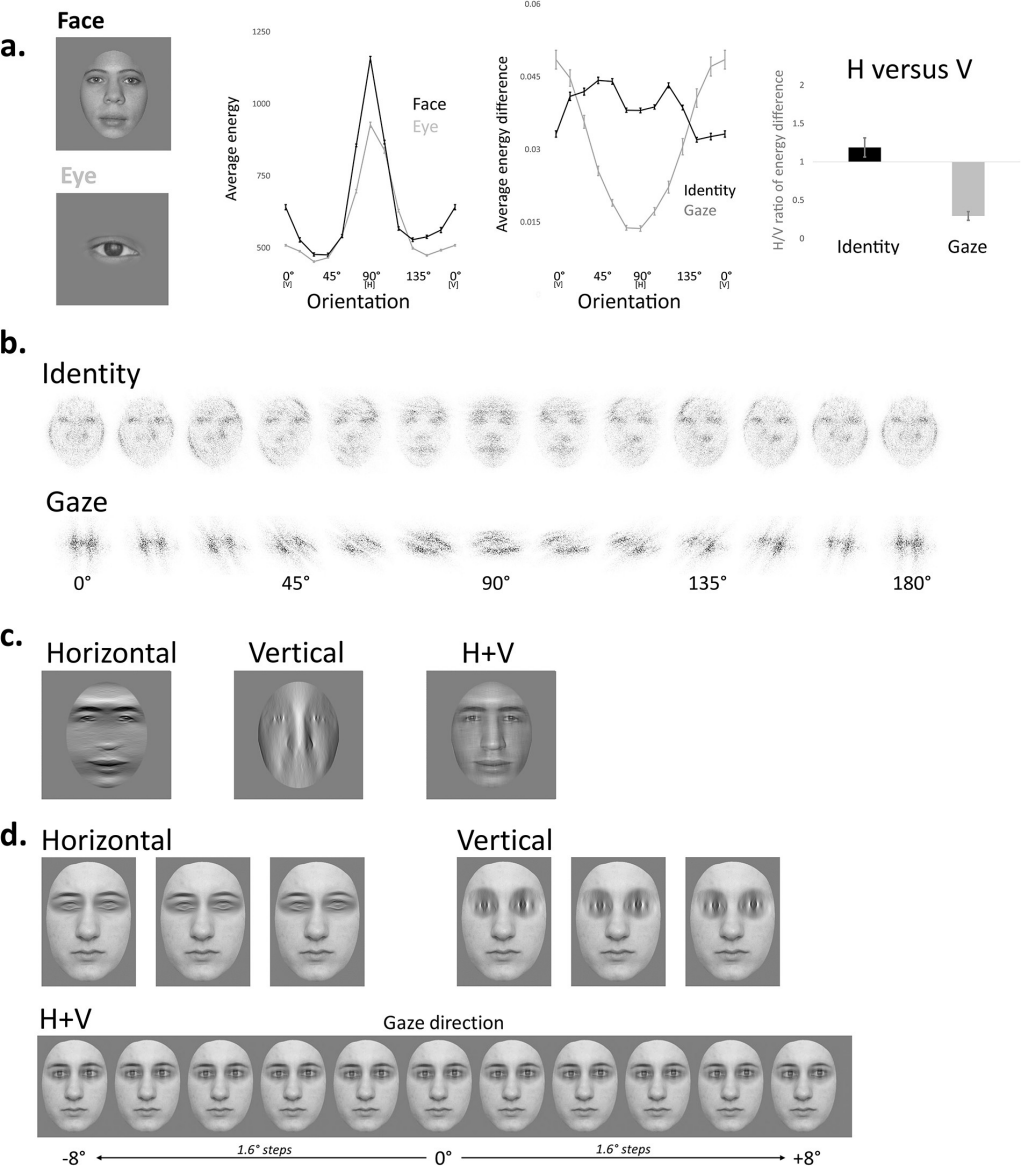
Image analyses (see details in S1 File) and stimuli. a. Examples of images analyzed to estimate the energy distribution of identity and gaze cues. The left plot shows the distribution of total image energy averaged across SF in 15°-wide orientation ranges. Both the face (black line) and the eye region (grey line) contain most energy in the horizontal range. The middle plot depicts the average differences in energy that result from variations in face identity (black line) and from gaze shifts (grey line). Identity energy differences are broadly tuned to horizontal orientation, whereas lateral gaze shifts disproportionately modulates the vertical components of the eye stimulus. The right graph shows the ratio between the average energy differences in horizontal and vertical for variations in identity (black bar) and gaze (grey bar) and further illustrates that variations in identity and gaze differentially modulate horizontal and vertical component of the stimulus image. b. 2D illustration of the normalized average energy differences related to identity and gaze variations depicted in a (see details in S1 File). c. Illustrative examples of the egg-shaped and orientation-filtered face stimuli used in the identification experiment. d. Examples of the stimuli used in the gaze categorization task. Filtered eye regions were pasted onto a stable full spectrum face context. The eleven gaze directions are illustrated below in the horizontal+vertical condition.

While the human face contains most of its contrast (or energy) in the horizontal range (Fig 1a; see details on image analyses in S1 File), identity variations alter image energy over a broader range of orientations (Fig 1b; see [21, 22] for similar evidence). However it is only in ranges close to horizontal that such energy differences encompass the main facial features (e.g., eyes, eyebrows) and their top-heavy asymmetric configuration (Fig1a and 1b; [7, 21, 23]), i.e., the cues relevant to high-level face identification [24–26]. These peculiarities in the structure of the face stimulus may confer to its horizontal content the role of a gateway, determining access to any type of facial information, in favour of a fixed/domain-specific horizontal tuning of human face perception.

However not all facial cues are horizontally-structured and face perception may flexibly tune to the orientation carrying the task-relevant input (as reported in the spatial frequency and feature domains; [27, 28]). Indeed, while the eye region maximally conveys energy in the horizontal range, lateral shifts of gaze direction modulate image energy in the vertical range (Fig 1a; [29]). In order to determine the fixed versus flexible nature of the orientation tuning for face perception, the present work addressed whether the mechanisms recruited to compute gaze direction attune to the vertical energy profile of gaze direction cues, marking a flexible orientation tuning, or whether they are horizontally-tuned, confirming the fixed gateway nature of horizontal information in face processing.

In order to derive a fine measure of sensitivity to gaze we presented pictures of a face staring at 11 close locations on the horizontal plane, evenly distributed around direct gaze. Participants were asked to judge gaze direction as being direct, averted to the left, or to the right. By fitting individual response curves, we assessed the sensitivity to averted gaze shifts across cardinal (i.e. horizontal and vertical) orientations. We further derived a measure of sensitivity to direct gaze by computing the so-called cone of direct gaze (COD), namely the range of gaze deviations being reported as direct [30–33]. Participants additionally performed an identification task on upright and inverted faces. Across tasks, the processing of gaze and identity cues was restricted to the horizontal range, the vertical range, or their combination; the latter condition served as a baseline.

## Materials and methods

### Participants

Twenty-five students (UC Louvain, Belgium) gave their written informed consent to take part in the experiment in exchange for monetary compensation (8 euros per hour of testing). Since the total experiment lasted 2 hours, each participant received 16 euros. They were 20 females and 5 males, aged 24 (± 5.6) on average (2 participants were left-handed). Optical corrections were used when necessary. Participants had normal vision as verified by a Landolt C acuity test and scored within the normal range on standard tests of astigmatism. The experimental protocol adhered to the Declaration of Helsinki and was approved by the local ethical committee (Psychological Sciences Research Institute, UC Louvain). All participants except one performed two experimental tasks: identification and gaze direction judgment.

### Stimuli

Across experiments, stimuli contained either the horizontal (H), vertical (V), or both horizontal and vertical (HV) content of the image.

#### Identity experiment

The identity experiment was adapted from Goffaux and Dakin [8] experiment 1. Stimuli were twenty full-front photographs of unfamiliar Caucasian faces (half females) posing in a neutral expression and staring at the camera (image size: 256 x 256 pixels). Using Adobe Photoshop we embedded each face in an egg-shaped aperture to mask external cues to identity (facial outline, hair, neck and ears) and restrict identity processing to inner facial features, and normalized to obtain a mean luminance of 0 and a root-mean square (RMS) contrast of 1. Images were filtered using 20°-wide wrapped Gaussian filters centred on either horizontal (H), or vertical (V) range (see details in [7, 8]; Fig 1c).

#### Gaze experiment

In the gaze experiment, participants judged the gaze direction of centrally presented face stimuli presented one by one at screen centre. We selected one young adult Caucasian model from the stimulus database of Vida and Maurer [31]. The model had been photographed with a neutral expression while starring at positions ranging from 0° to 8.0° to the left and right of the camera lens, in five steps of 1.6° (see details on camera shooting procedure in [31]), making up a total of 11 gaze directions. We extracted the eyes and brows of the gaze images in Adobe Photoshop using a Gaussian contour selection (10 pixel progressive selection). Luminance and RMS contrast were matched between left and right halves of the eyes/brows image, and across the eleven gaze directions.

In Matlab 8.3.0.532 (R2014a), the 11 eyes/brows (one for each gaze direction) were pasted on a grayscale picture of a different, so-called ‘base’, face (image size: 313 x 428 pixels). The base face was of different identity but same gender as the gaze model. The base model posed full front, with a neutral expression, and fixated the camera lens. The original eyes/brows of the base face had been *a priori* removed using the patch tool in Adobe Photoshop, and hair, ears and neck were cropped. Pasting the eye gaze directions on a fixed full spectrum base model ascertained that participants judged gaze direction solely based on eye region and that any performance difference across orientation ranges reflected the orientation tuning of gaze processing and not of the entire face. Background luminance was set to grey (0.5 luminance across RGB channels).

The eleven resulting compound images were normalized to obtain a mean luminance of 0 and a root-mean square (RMS) contrast of 1. Next, they were fast Fourier transformed, and the resulting amplitude spectra were multiplied with wrapped Gaussian filters (with a standard deviation of 20°) centred on vertical (0°), horizontal (90°), or both vertical and horizontal angles. Filtering the compound stimuli (gaze direction varying on a stable base face) rather than the full original gaze stimuli further warranted that across-orientation modulations were related to gaze direction change only. Eyes and brows of the filtered compound images were selected again in Adobe Photoshop using the Gaussian selection tool (same procedure as above) and repasted on the unfiltered base face image (Fig 1d).

In both experiments, Horizontal+Vertical (H + V) stimuli were built by summing the H and V filtered images. After inverse-Fourier transformation, the luminance and root-mean square (RMS) contrast of all resulting images were adjusted to match mean luminance and contrast values of the original image sets (i.e., prior to filtering).

Identity and gaze stimuli were displayed against a grey background (0.5 luminance across RGB channels) using Eprime 2.0 at a viewing distance of 57 cm on a Viewpixx monitor (313 X 428 pixels, refresh rate: 75Hz, pixel size: 1920x1080). Identity stimuli subtended 6° (width) by 8° (height) of visual angle and gaze stimuli were 7.2° by 10°. In each experiment, all stimuli had equal luminance and RMS contrast.

### Procedure

Participants were seated in a dimly lit room in front of the computer screen with their head on a chinrest. They were instructed to fixate the centre of the screen. They performed the two experiments in a pseudorandomized order. The procedure employed in each experiment was adapted from past publications addressing the orientation dependence of identity processing [8] and human sensitivity to gaze [31].

#### Identity experiment

In the identity experiment, participants were instructed to respond as accurately as possible, using the computer keyboard, whether the pair of stimuli were the same or different. On 50% of trials stimuli differed, on 50% they were identical. A trial commenced with the presentation of a central fixation cross (duration range: 1250–1750 ms). The first stimulus then appeared for 700 ms, immediately followed by a 200-ms mask (256 × 256-pixel Gaussian noise mask; square size of 16 × 16 pixels). Both the first stimulus and the mask appeared at randomly selected screen locations across trials (by ± 20 pixels in x and y dimensions). Along with the inclusion of the mask, spatial jittering prevented participants from basing their decisions on a fixed and restricted region of the stimuli. After a 400-ms blank interval, where only the fixation marker was visible, the second stimulus appeared and remained visible until subjects made a response (maximum duration: 3000 ms).

In a given trial, faces belonged to the same filter-orientation or planar condition (i.e., both H, both V, or both H + V; both upright, or both inverted). Upright and inverted trials were clustered in 20-trial mini-blocks; the order of the other conditions (filter-orientation and similarity) was random. There was a 10-s resting pause every ten trials. Feedback (a running estimate of accuracy) was provided every 60 trials. Prior to the experiment, subjects practiced the task over 70 trials. There were 12 conditions in a 2 × 2 × 3 within-subject design. The three factors were: similarity (same versus different), planar-orientation (upright versus inverted), and filter-orientation (H, V or H + V). We ran 40 trials per condition, to give a total of 480 experimental trials.

#### Gaze experiment

In the gaze experiment participants had to report as accurately as possible whether each face stimulus stared to the left, to the right, or straight at them (direct gaze), using the left, right, or down arrow keys, respectively. A trial started with the central presentation of a fixation cross for a variable duration (randomly varying between 750 and 1000 ms). Then the stimulus appeared at the center of the screen for 400 ms followed by a 200-ms Gaussian white noise mask (block size: 15 x 15 pixels; size: 315 x 435 pixels) of the same global luminance and contrast as the gaze stimuli. A dark grey cue ‘REPONSE’ (response in French) then appeared and stayed on screen until the response was given. Self-paced resting pauses occurred every 55 trials during which participants received feedback about their average accuracy over the last block.

Gaze direction (11 directions) and image orientation content (H, V, and HV) varied randomly from trial to trial and each gaze direction was equally likely. There were two experimental sessions on different days. In each session, participants performed 990 trials (60 trials per conditions; total of 1980 trials).

Following task instruction participants completed 12 practice trials with the full spectrum (non-orientation-filtered) versions of a different model of opposite sex than the one used during the experimental trials. They received feedback after each practice trial in the form of a green ‘CORRECT’ or a red ‘ERREUR’ (error in French) central label. Next, they performed 9 practice trials with orientation-filtered versions of the practice stimulus to get familiarized to orientation filtered images. During practice, participants were presented with extreme left and right gaze directions (-8° and +8°) as well as direct gaze. Participants had to reach an overall accuracy of 85% during practice in order to proceed to the experimental trials.

### Data analyses

In the identity experiment, we estimated individual sensitivity (d′) based on hits and correct-rejections in every planar-orientation and filter-orientation condition, following the loglinear approach [34]. We submitted the sensitivity measures to a 2 × 3 repeated-measure ANOVA. Conditions were compared using two-tailed paired t tests at a Bonferroni-corrected alpha value of .0042 (for the 12 comparisons conducted).

Next, we computed an individual horizontal tuning index for upright and inverted faces separately by subtracting d’ in V condition from d’ in H condition. As expected we observed that a majority of participants had a strong H tuning index larger than 0 (21 out of the 24 subjects tested in the identity experiment) when faces were upright; H tuning was comparably weaker and less frequent (14 out of the 24 subjects) when faces were inverted.

To address whether H tuning observed for upright face identification generalizes to gaze direction processing, we analysed the gaze direction performance of the 21 subjects who performed the identification task and showed a significant H tuning for upright identification.

Individual datasets were compiled across the two sessions of the gaze experiment. Logistic functions were fitted to the proportion of ‘left’ and ‘right’ responses as a function of stimulus gaze direction using the Matlab 8.3.0.532 (R2014a) glmfit as done e.g. in Vida and Maurer (2012). The selectivity or sharpness of averted gaze direction processing was estimated by the logistic slope coefficient.

In line with past studies (e.g., [31]), we computed the inverse of the summed logistic functions fitting “left” and “right” psychometric curves to derive the “direct” response function. We estimated its peak (i.e., the deviation most likely to be perceived as being direct) and its width, the so-called cone of direct gaze, by computing the distance (in degrees of gaze deviation) separating the points of intersection of each logistic function with the “direct” function. All fitting was conducted at the individual level.

We addressed the orientation dependence of gaze direction processing by comparing the parameter estimates of the “left”, “right”, and “direct” psychometric fits across orientations using one-way repeated measure ANOVAs with Orientation content as a within-subject factor (3 levels: H, HV, V). Conditions were compared using two-tailed paired t tests at a Bonferroni-corrected alpha value of .0055 (for the 9 comparisons conducted).

To compare the orientation dependence of human sensitivity to gaze and identity, we computed the Hedges’ g size of the performance difference across orientations (i.e., the difference between the means divided by the pooled standard deviation; [35]). Orientation dependence is quantified as the number of standard deviations separating the mean performance across orientations. In the identity task, we first subtracted inverted from upright performance per individual in order to evaluate the inversion effect, i.e. upright face-selective identification. Then we computed the Hedges’ g for each orientation comparisons. The size of orientation differences in sensitivity to left, right, and direct gaze (i.e., logistic slope coefficient and cone of direct gaze) was evaluated the same way.

## Results

### Face identification across cardinal orientations

**Fig 2:**
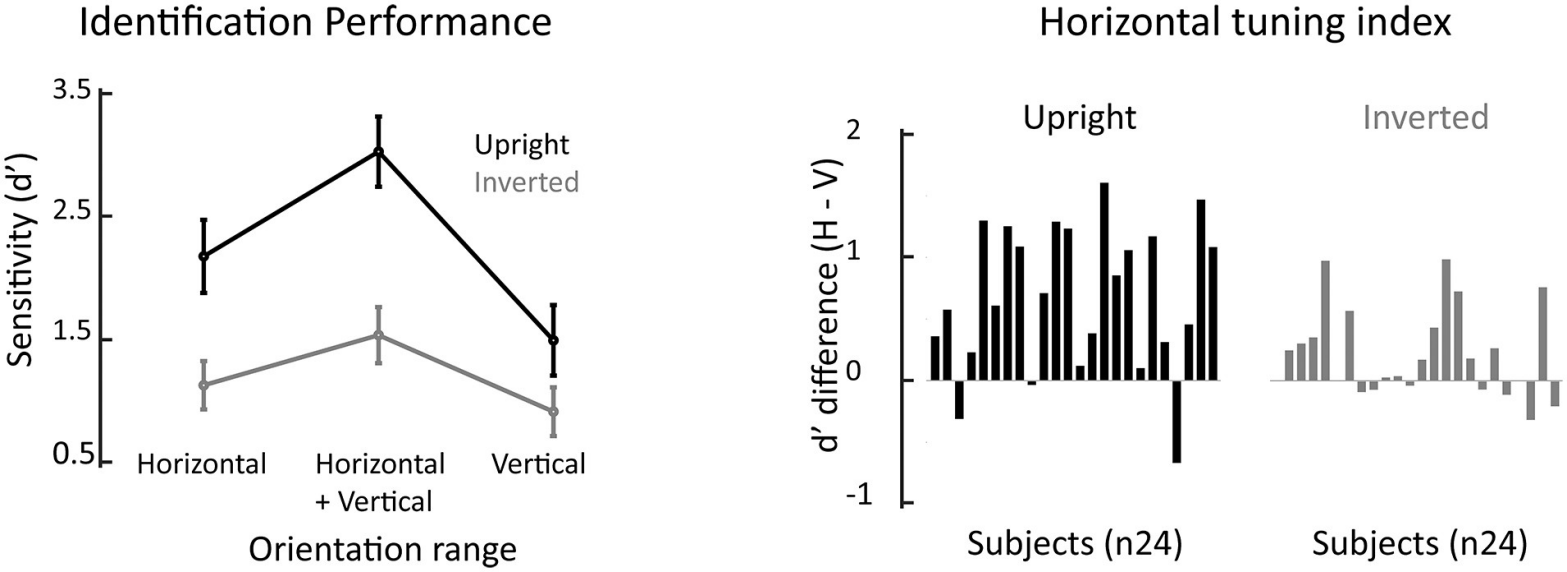
Face identification performance. Left. Group-averaged sensitivity (d′) per planar orientation (upright versus inverted) and filter-orientation (horizontal, horizontal+vertical, vertical). Error bars are ± 95% confidence intervals. Right. Individual horizontal tuning index (horizontal minus vertical d’) for upright and inverted faces separately.

The group-averaged (n = 24) identification performance is plotted on Fig 2. Table 1 details sensitivity mean and 95% confidence interval for each Orientation content and Planar Orientation condition.

**Table 1:** Face identification performance in sensitivity d’ (n = 24; means and 95% confidence intervals).

N = 24	Horizontal		Horizontal+Vertical		Vertical	
	Mean	95% CI	Mean	95% CI	Mean	95% CI
Upright	2.17	[1.86 2.48]	3.02	[2.73 3.31]	1.49	[1.2 1.78]
Inverted	1.13	[0.92 1.33]	1.53	[1.3 1.77]	0.91	[0.71 1.11]

Sensitivity in the identification task was significantly influenced by the orientation content and planar orientation of the face stimuli (see statistic details in Table 2). As expected, the interaction between these factors was significant.

**Table 2:** Summary of F statistics (n = 21).

**Sensitivity to Identity** **Two-way ANOVA**	**F statistic**	**p value**	**partial η**^**2**^
Orientation Content	65.43	0.00001	0.74
Planar orientation	168.65	0.00001	0.88
Orientation x PlanarOrientation	23.13	0.00001	0.5
**Sensitivity to Gaze** **One-way ANOVA**	**F statistic**	**p value**	**partial η**^**2**^
"Right" slope	33.1	0.0001	0.62
"Left" slope	66.96	0.0001	0.77
"Direct" Peak	0.15	0.85	0.01
"Direct" Cone	3.2	0.052	0.15

When faces were upright, the main effect of Orientation content was substantial (F(2,46) = 61.97, p< .0001, partial η^2^ = .73). Sensitivity to identity decreased significantly from HV to H and from H to V condition (two-tailed Bonferroni corrected t-test: ps< .0001). When faces were inverted, the main effect of Orientation content was still robust but attenuated compared to upright condition (F(2,46) = 25.414, p< .0001, partial η^2^ = .52). Sensitivity for HV stimuli surpassed sensitivity for H and V (two-tailed t-test, with a Bonferroni-corrected alpha value of .0042: ps< .0006), but was comparable between the latter conditions (two-tailed t-test: p = .01).

Inversion impaired sensitivity in all orientation ranges (one-tailed Bonferroni corrected t-test: ps< .0001); when compared across orientation ranges the magnitude of the inversion effect was significantly stronger in HV, next in H, and last in V (two-tailed Bonferroni corrected t-test: HV-H: p< .002, Hedges’s g: .78; HV-V: p< .00001, Hedges’s g: 1.24; H-V: .0004, Hedges’s g: 0.82).

The finding that sensitivity to upright, but not inverted, face identity is highest in the H than the V range of face information agrees with past evidence (e.g., [9, 11]). A robust H tuning was found in 21 out of the 24 participants for upright face identification (see Fig 2); in contrast H tuning was weak and less frequent for inverted face identification.

Since the present work addresses whether H tuning generalizes to any task involving upright faces, the subsequent analyses were conducted on the sample of participants showing the expected H tuning for upright face identification, excluding the 3 participants showing the reverse tuning for upright face identification.

### Sensitivity to gaze direction across cardinal orientations

**Fig 3:**
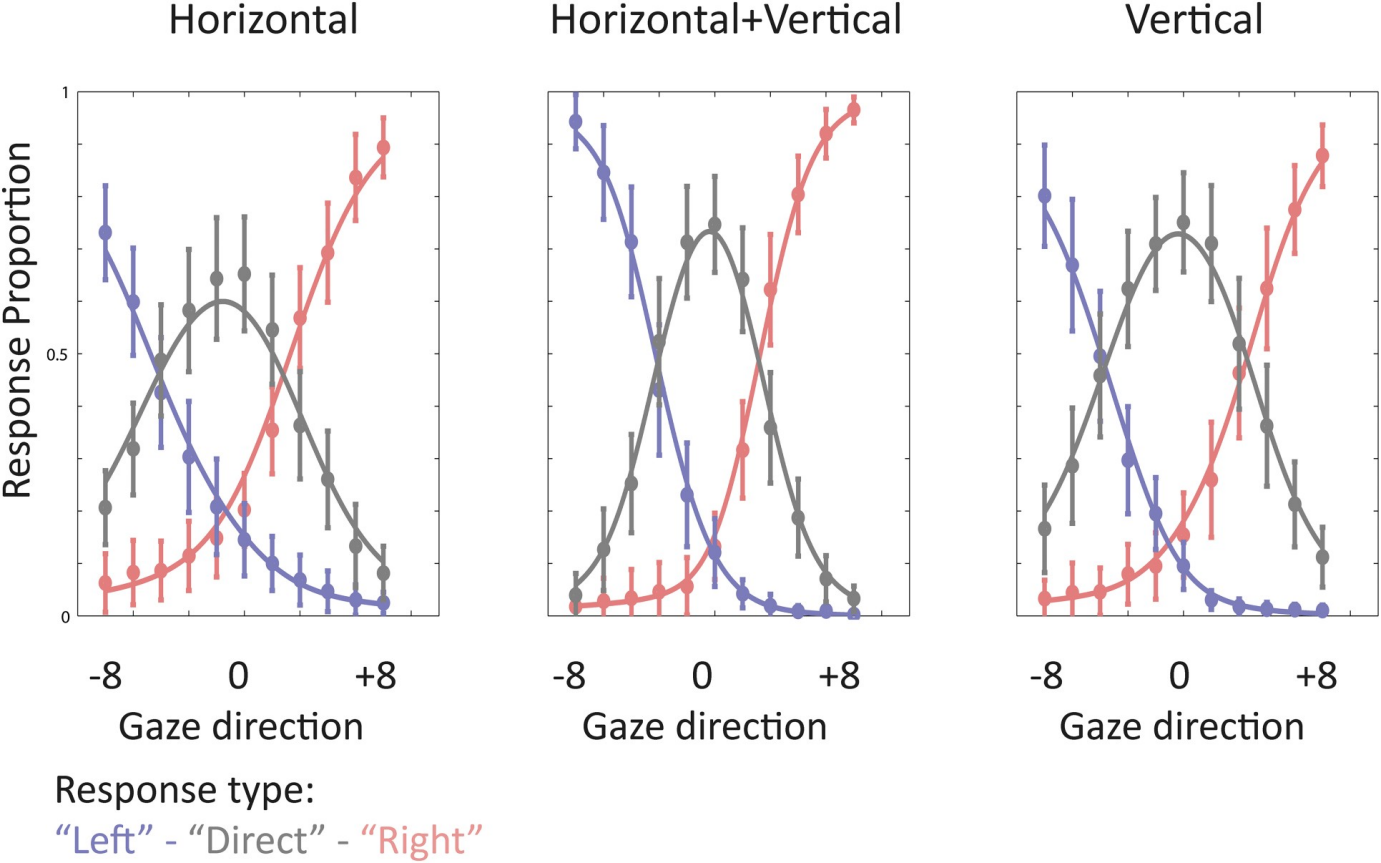
Gaze categorization performance. Performance is plotted separately for each orientation condition. “Left”, “right”, and “direct” responses are coded in blue, grey, and red colors, respectively. Dots depict group-averaged response proportions (n = 21). Error bars are ± 95% confidence intervals. “Left” and “right” lines are the logistic fits to group-averaged “left” and “right” proportions of responses. The “direct” line results from the subtraction of the summed logistic functions from 1.

Fig 3 illustrates the group-averaged performance in the gaze categorization task. The proportions of “left” and “right” responses were fitted by negative and positive logistic functions, respectively. The function fitting the proportion of “direct” responses was obtained by subtracting the sum of the “left” and “right” logistic functions from 1.

**Fig 4:**
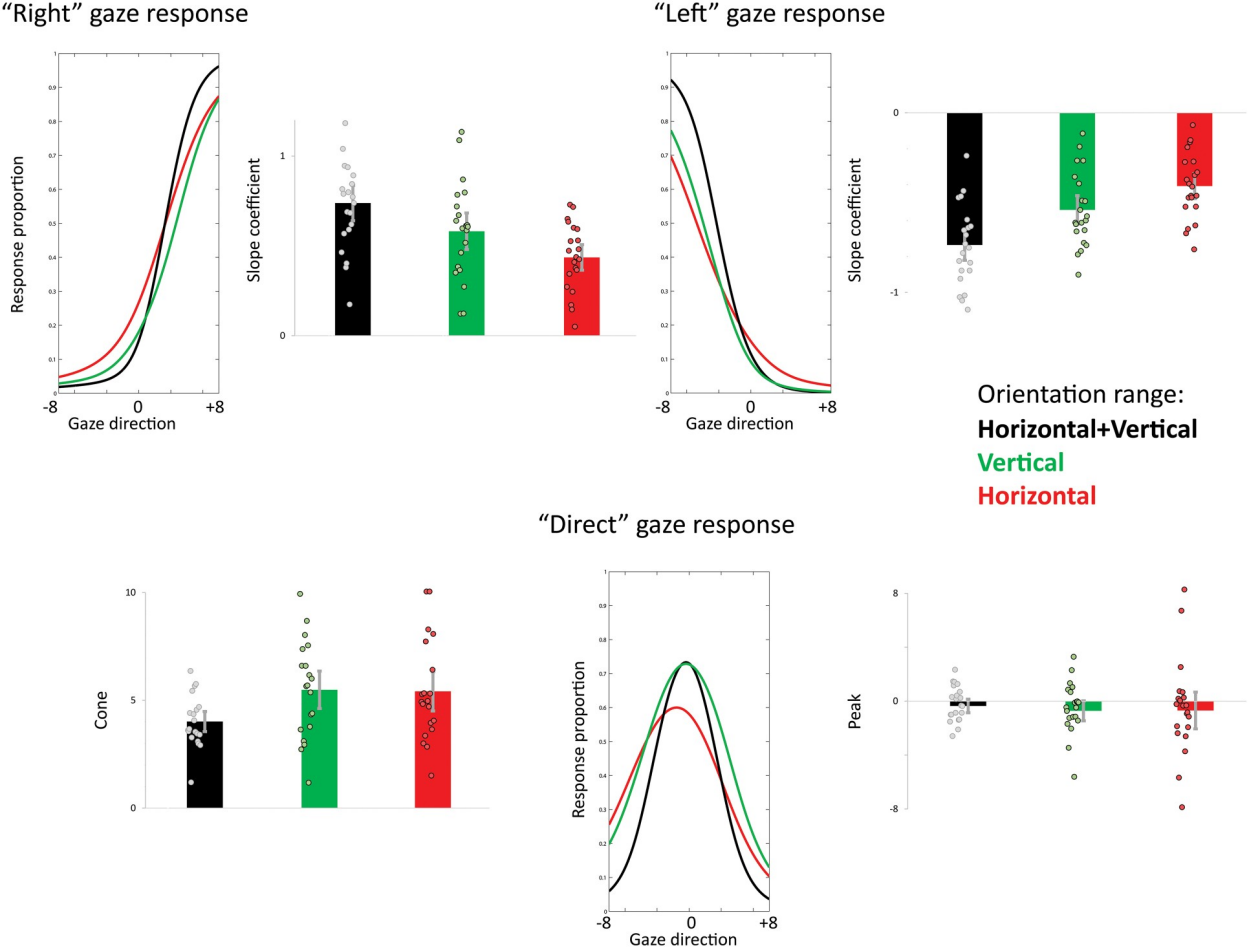
Parameter estimates of gaze categorization performance across orientations. Top. Logistic fits estimates of slope steepness for the “left” and “right” responses (top). Bottom. Estimates of the peak and width (i.e., cone of direct gaze) of “direct” gaze responses. Line graphs represent the same group-averaged (n = 21) fits to “left”, “right”, and “direct” psychometric curves (as shown on Fig 3) compared across orientations. The bar graphs depict group-averaged parameter estimates and error bars are the ± 95% confidence intervals. Dots are individual data points.

To estimate the sensitivity to averted and direct gaze we measured the sharpness of the function relating the proportion of averted (“left” and “right”) and “direct” to the actual gaze direction presented in each individual and orientation range. Sensitivity to gaze was estimated based on the logistic slope coefficients for averted “left” and “right” likelihood and the size (in gaze deviation degrees) of the cone of direct gaze for “direct” gaze response. The means and 95% CIs of the parameter estimates are provided in Table 3. Fig 4 compares the logistic and the derived DG functions across orientations.

**Table 3:** Slope coefficients of “left” and “right” psychometric function, cone and peak parameters of the “direct” psychometric function. Mean and 95% confidence intervals (n = 21).

	Horizontal		Horizontal+Vertical		Vertical	
	mean	ci	mean	ci	mean	ci
"Right" slope	0.44	[0.36 0.51]	0.74	[0.64 0.84]	0.58	[0.48 0.68]
"Left" slope	-0.41	[-0.48–0.34]	-0.74	[-0.82–0.65]	-0.54	[-0.62–0.46]
"Direct" Cone	5.42	[4.5 6.33]	4.02	[3.55 4.48]	5.49	[4.62 6.36]
"Direct" Peak	-0.69	[-2.06 0.67]	-0.36	[-0.86 0.15]	-0.72	[-1.46 0.02]

Repeated-measure one-way ANOVAs were performed for slope coefficients, peak and cone estimates with Orientation content as a within-subject factor (see Table 2 for a summary of the statistics).

Orientation content significantly modulated the slopes of the logistic functions fitting “left” and “right” response proportions. We tested for a potential effect of response type on the slope steepness by submitting the absolute ‘left’ and ‘right’ slope values to a repeated-measure ANOVA testing for the effect of Orientation content, and Response type (‘left, ‘right’). The main effect of Orientation content was the only significant effect (F(2,40) = 65.76, p< .00001, partial η^2^ = .77). Response type did not significantly influence slope values (main effect of Response type: F(1,20) = 1.73, p = .2, partial η^2^ = .08); it also did not modulate the effect of Orientation (Orientation content by Response type interaction: F(2,40) = .555, p = .58, partial η^2^ = .03).

Orientation content only marginally modulated the width of “direct” gaze response function (i.e., the cone of direct gaze); it did not influence its peak.

Slope coefficients and cone of direct gaze were compared across orientations by means of two-tailed paired ttests at a Bonferroni-corrected alpha value of .00555 (Table 4). The slopes of “left” and “right” response functions were steeper in HV compared to H and V, suggesting a finer sensitivity to gaze direction when cardinal orientations are combined. Interestingly we also found both slopes to be steeper in V compared to H.

**Table 4:** Summary of t statistics and Cohen d effect size (n = 21).

Corrected alpha: .0055		t statistic	p value	degree of freedom	CI	Cohen d
"Right" slope	H vs V	-3.72	0.0014*	20	[-0.23–0.06]	-0.61
	HV vs H	8.65	0.00001*	20	[0.23 0.38]	1.12
	HV vs V	4.2	0.0004*	20	[0.08 0.23]	0.58
"Left" slope	H vs V	3.99	0.0007*	20	[0.06 0.2]	0.65
	HV vs H	-12.24	0.00001*	20	[-0.38–0.27]	-1.26
	HV vs V	-7.89	0.00001*	20	[-0.25–0.14]	-0.83
"Direct" cone	H vs V	-0.12	0.9058	19	[-1.32 1.17]	-0.03
	HV vs H	-2.57	0.02	18	[-1.84–0.19]	-0.72
	HV vs V	-2.43	0.025	18	[-2.22–0.16]	-0.77

The gaze deviation at which the proportion of “direct” reports culminated was close to 0°, with a negligible leftward bias, and did not differ across orientations. The cone of direct gaze only marginally differed between HV and the other orientation ranges. It was similar between H and V.

### Do identity and gaze rely on orientation to a comparable extent?

The above results indicate that human sensitivity to identity and gaze tune to distinct cardinal orientations, horizontal and vertical, respectively.

**Fig 5:**
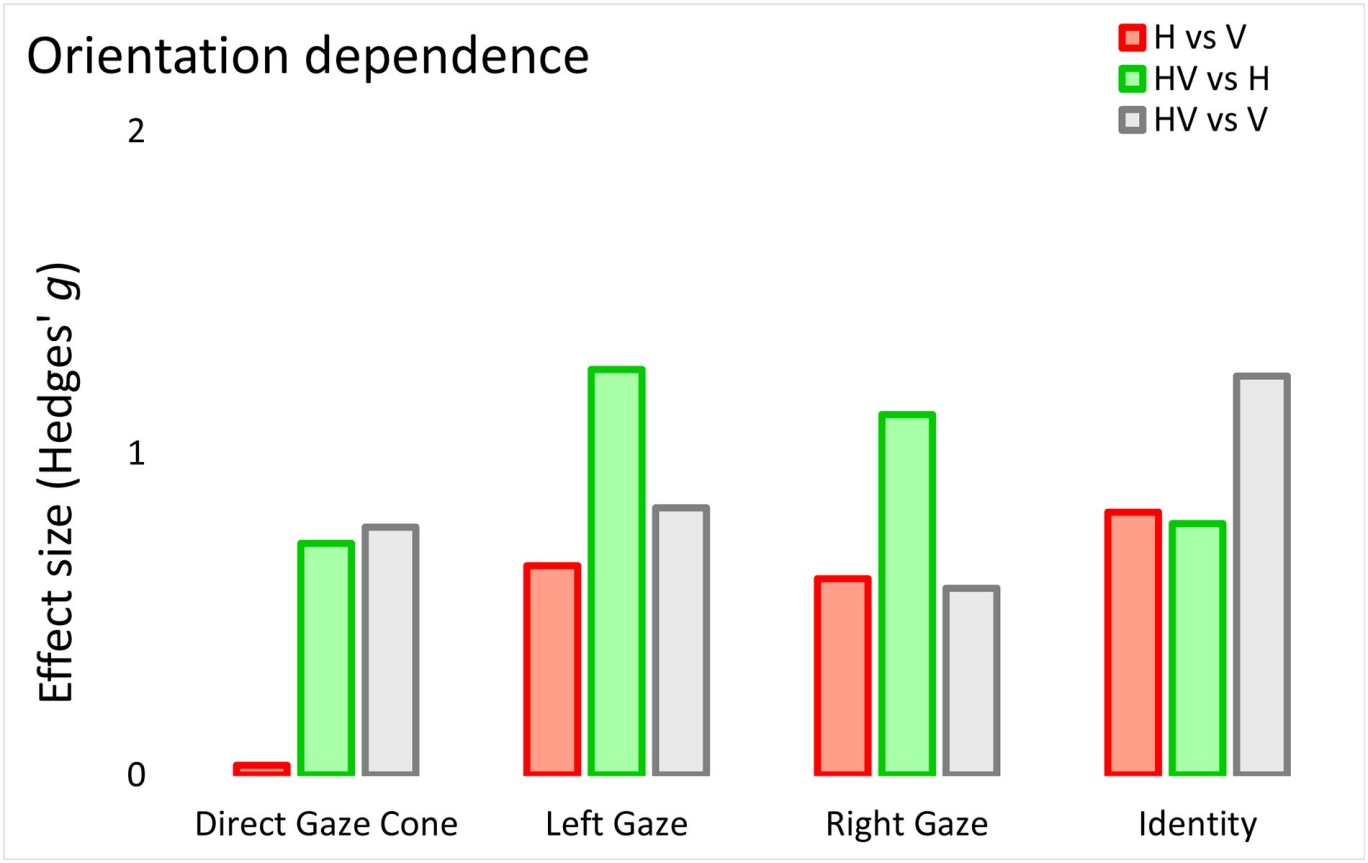
Size of the orientation dependence in gaze and identity experiments. We computed the Hedges’ g of the across-orientation differences for left and right gaze psychometric slope coefficients and for the cone of direct gaze; for identity, across-orientation difference was computed on the size of the face inversion effect (upright minus inverted).

We wanted to compare orientation dependence more directly across tasks using a common metric. We computed the size of the across-orientation difference in face inversion effect, slope coefficients, and cone of direct gaze using Hedges’ g. Overall, orientation differences in selectivity were of comparable magnitude across identity and gaze experiments, except for the H versus V direct gaze cone difference, for which no significant difference was found. Interestingly, it seems that for identification adding H input to V information (i.e., HV versus V comparison; grey bar on Fig 5) for gaze processing led to a comparable gain in performance as adding V input to H information (i.e., HV versus H comparison; green bar on Fig 5). This indicates that the combination of orientations facilitated processing of identity and gaze in an unspecific way, through the accumulation of more information.

## Discussion

The human face is a horizontally-structured stimulus, and the processing of identity is tuned to the horizontal range. In contrast, gaze shifts are mostly represented in the vertical range. We investigated whether the face processing system flexibly tunes to vertical information when processing gaze direction, or whether it invariantly relies on the horizontal range, supporting the domain specificity of orientation tuning for faces and the gateway role of horizontal content to access face information. Participants judged the gaze direction of faces staring at a range of lateral positions. Stimuli selectively revealed the horizontal (H), vertical (V), or combined (HV) content of the eye region. The participants additionally performed an identification task with orientation-filtered upright and inverted face stimuli. Performance in both tasks was best when the stimuli combined horizontally- and vertically-oriented content. This is not surprising considering the fact that information was richer in this condition.

Most participants identified faces better based on horizontal than vertical information confirming the horizontal tuning of face identification. Horizontal tuning was strong for upright faces, and substantially attenuated under the influence of inversion. These results confirm past evidence that the horizontal tuning of face identification is not passively inherited from low-level encoding stages tied to physical properties of the face image, but emerges at a high-level stage where upright and inverted faces employ distinct processing chains ([11, 16, 18]; see also our image analyses).

The same individuals who showed a horizontally-tuned sensitivity to upright face identification revealed a vertically-tuned sensitivity to gaze direction on average. The psychometric functions fitting the “left” and “right” response proportions as a function of gaze direction were indeed steeper when based on vertical than on horizontal information. In other words, the mechanisms involved in the fine perception of averted gaze direction were most sensitive when relying on the vertical information present in the eye region. The finding of a vertically-tuned processing of gaze direction favours the hypothesis that visual encoding of face information flexibly switches to the orientation channel carrying the cues most relevant to the task at hand. Several authors proposed that simple low-level mechanisms coding the iris/sclera luminance configuration may be sufficient to determine gaze direction ([29, 36–38] but see [39] for adaptation evidence pointing to a high-level locus). The finding that our participants preferentially relied on the vertical range where image energy fluctuations were the most prominent supports the viability of such a low-level mechanism. More generally, it suggests that the orientation tuning of identity and gaze processing may emerge at different (high and low, respectively) levels of visual processing.

While participants matched whole face identity in the identity task, they were explicitly instructed to focus on the eye region in the gaze task. The differential reliance upon cardinal orientations across these tasks may therefore stem from differences in the spatial extent of the task-relevant information (i.e., whole face identity versus local gaze direction). In S1 Fig and S2 File, we provide evidence that face identification preferentially relies on horizontal cues even when based on the local analysis of the eye region. This suggests that the identity processing is horizontally-tuned, irrespective of the global versus local nature of the task and stimuli.

In order to challenge the domain-specific hypothesis, we deliberately concentrated our investigation on the perception of lateral gaze shifts, which are conveyed by the vertical structure of the eye region. Humans are most sensitive to lateral gaze shifts [31, 33]. However, gaze also shifts in vertical (up/down) direction, resulting in energy fluctuations in the horizontal range (image analyses not shown here). It is likely that the processing of vertical gaze shifts preferentially relies on orthogonal horizontal input.

While the sensitivity to averted gaze shifts was sharper for vertical than horizontal stimuli, sensitivity to direct gaze as indexed by the peak and width of the distribution of ‘direct’ gaze responses, namely the cone of direct gaze, did not disclose any preference for horizontal or vertical oriented information. Instead the so-called cone of direct gaze (COD) increased comparably in each range compared to the condition where orientations were combined (HV). The values reported here matched those previously reported indicating the validity of our measures (range from 5 to 7.5°; see review in [33]). Past studies further showed that humans have a prior expectation that another’s gaze is directed towards them with stimulus uncertainty increasing the COD [40, 41]. The broadening of COD we observed here in horizontal and vertical ranges may reflect the increase in stimulus uncertainty and, therefore, the increased reliance on direct gaze priors.

When put on a common metric, orientation dependence was comparable in strength for gaze and identity encoding (Fig 5). This suggests that the predominant horizontal structure of the eye region and of the surrounding face did not mitigate the flexible tuning to vertically-oriented gaze cues. A closer look at the conditions where horizontal and vertical information was combined is informative with this respect. If the horizontal structure of the face stimulus were to provide a privileged gateway to any type of facial cue, it should strongly facilitate the processing of vertical gaze cues, whereas providing vertical information along with horizontal input should only minimally improve identification performance. Contradicting this view, we observed comparable performance benefits suggesting that the absolute predominance of horizontal information in the face stimulus and the eye region did not interfere with the preferential reliance on vertical cues when processing gaze.

The past and present findings suggest a relative preservation of primary features like orientation at high-level levels of visual processing. The mechanisms empowering such preservation are unclear. Since the present behavioural evidence only reflects the final output of a complex visual processing chain, we cannot rule out that early stages of gaze processing are invariably driven by the predominant horizontal structure of the face and the eye stimulus. As proposed by Dakin and Watt [7], the face stimulus and its features may be initially signalled via their massively horizontal structure, which would serve as an index for further processing of e.g., identity, gaze direction, emotion. Alternatively, the different orientation ranges coded in primary visual cortex may project onto different high-level regions for further processing with horizontal signals preferentially targeting ventral regions responsible for identity coding and vertical signals reaching dorsal regions involved in the coding of gaze direction. With this respect it is interesting to note that electrophysiological recordings recently indicated the co-existence of vertically- and horizontally-tuned face-selective regions within the monkey inferotemporal cortex [42]. Addressing the spatiotemporal dynamics of orientation tuning and integration along the ventral (and dorsal) visual pathways of the human brain will be the goal of our future investigations.

In natural settings human observers simultaneously sample different types of face information (e.g., gaze, expression, identity, etc.). In the present experiment, participants exclusively focused on the gaze direction of a single identity (in line with past studies using a similar design; e.g., [31]). There are only a few studies addressing the mutual influence of gaze and identity processing (e.g., [43–45]). How the visual system prioritizes selective ranges of orientation in these parallel processing conditions should also be further investigated. Considering the coarseness, i.e., the predominantly low spatial frequency content, of vertically-oriented gaze cues (S2 Fig), their processing at distance and/or in the periphery may be exceptionally fast and efficient.

Even though identity and gaze are likely processed in parallel in everyday life, their processing has been proposed to rely on separable mechanisms and representations [39, 46]. The distinct orientation dependence profiles for identity and gaze processing we report here further supports such functional separation. Gaze is a core visual cue that governs social communication among humans. The fact that it is carried by basic stimulus properties (i.e., energy fluctuations) within an orientation range orthogonal to the one relevant for identity may be particularly advantageous at a computational level.

## Conclusions

The present findings suggest that the predominant horizontal structure of the face stimulus is not a mandatory gateway of face processing. Instead, they show that the orientation tuning of human face processing flexibly switches to the orientation range carrying the most relevant cues for the task at hand. These findings open new exciting perspectives towards a better understanding of feature preservation along the visual pathway.

## Supporting information

S1 Fig Sensitivity to local identity variations. A. Illustration of the congruent-same (i.e., fully identical faces) and different-incongruent (i.e., identity differences restricted to the eye region) pairs of the congruency paradigm (adapted from Fig 7 in [8]). B. Group-averaged sensitivity to identity differences restricted to the eye region. Error bars are 95% confidence intervals.
(DOCX)

S2 Fig Orientation profile of gaze direction variations in low, medium and high spatial frequencies. Since the focus of this work is on the orientation dependence of identity and gaze processing, image analyses were performed but then averaged across spatial frequencies. Here the plot shows the average image energy differences resulting from variations in gaze directions (as plotted on Fig 1a, right plot) as a function of orientation selectively in the low, middle, and high SF bands of the eye image. This plots indicates that the vertical predominance of gaze direction cues is most notable in the low to mid SF. At high SF, gaze shifts the horizontal fine structure of the eye likely due to the slight displacement of the lower lid (see Fig 1b). Error bars are 95% confidence intervals. (see S1 File).
(DOCX)

S1 File Image analyses. We evaluated how stimulus variations in gaze direction and identity influenced image energy profile across orientations. For gaze direction, we delineated the eye region in the 11 full-spectrum images (one for each gaze direction), replaced all pixels outside this region by grey values, and cropped the image to fit eye region borders in order to capture energy variations selectively induced by gaze shifts. For identity, we analysed the egg-shaped 20 face identities used in the face identification experiment.
(DOCX)

S2 File Sensitivity to local identity variations. A notable difference between the identity and the gaze experiments described in the main manuscript is the spatial extent of the task-relevant cues. Gaze cues are by definition local and participants were instructed to focus on the eyes to categorize gaze direction. In contrast, in the identity experiment, participants had to match whole faces and identity variations extended to the whole face surface. To address whether face identity still best processed in the horizontal range when based on local identity cues, we re-analysed the behavioral data of the experiment 4 reported by Goffaux and Dakin (2010), in which participants were instructed to process faces locally.
(DOCX)

S3 File Raw data files. Eprime log files were merged across gaze experiment sessions.
(ZIP)

## References

[1] Maurer D, Grand RL, Mondloch CJ. The many faces of configural processing. Trends in cognitive sciences. 2002;6(6):255–60. . 12039607 10.1016/s1364-6613(02)01903-4

[2] Rossion B. Picture-plane inversion leads to qualitative changes of face perception. Acta psychologica. 2008;128(2):274–89. 10.1016/j.actpsy.2008.02.003 . 18396260

[3] Burton AM, Schweinberger SR, Jenkins R, Kaufmann JM. Arguments Against a Configural Processing Account of Familiar Face Recognition. Perspectives on psychological science: a journal of the Association for Psychological Science. 2015;10(4):482–96. 10.1177/1745691615583129 . 26177949

[4] Sekuler AB, Gaspar CM, Gold JM, Bennett PJ. Inversion leads to quantitative, not qualitative, changes in face processing. Current biology: CB. 2004;14(5):391–6. 10.1016/j.cub.2004.02.028 . 15028214

[5] Rhodes G, Brake S, Atkinson AP. What’s lost in inverted faces? Cognition. 1993;47(1):25–57. . 8482070 10.1016/0010-0277(93)90061-y

[6] de Valois R, de Valois K. Spatial Vision. Annual Review of Psychology. 1980;31:309–41. 10.1146/annurev.ps.31.020180.001521 7362215

[7] Dakin SC, Watt RJ. Biological "bar codes" in human faces. Journal of vision. 2009;9(4):2.1–10. 10.1167/9.4.2 . 19757911

[8] Goffaux V, Dakin SC. Horizontal information drives the behavioral signatures of face processing. Frontiers in psychology. 2010;1:143 10.3389/fpsyg.2010.00143 . 21833212 PMC3153761

[9] Goffaux V, Greenwood JA. The orientation selectivity of face identification. Scientific reports. 2016;6:34204 10.1038/srep34204 . 27677359 PMC5039756

[10] Pachai MV, Bennett PJ, Sekuler AB. The Bandwidth of Diagnostic Horizontal Structure for Face Identification. Perception. 2018;47(4):397–413. 10.1177/0301006618754479 . 29350095

[11] Pachai MV, Sekuler AB, Bennett PJ. Sensitivity to Information Conveyed by Horizontal Contours is Correlated with Face Identification Accuracy. Frontiers in psychology. 2013;4:74 10.3389/fpsyg.2013.00074 . 23444233 PMC3580391

[12] Balas B, Huynh C, Saville A, Schmidt J. Orientation biases for facial emotion recognition during childhood and adulthood. Journal of experimental child psychology. 2015;140:171–83. 10.1016/j.jecp.2015.07.006 . 26247810

[13] Duncan J, Gosselin F, Cobarro C, Dugas G, Blais C, Fiset D. Orientations for the successful categorization of facial expressions and their link with facial features. Journal of vision. 2017;17(14):7 10.1167/17.14.7 . 29228140

[14] Huynh CM, Balas B. Emotion recognition (sometimes) depends on horizontal orientations. Attention, perception & psychophysics. 2014;76(5):1381–92. 10.3758/s13414-014-0669-4 . 24664854 PMC4096061

[15] Yu D, Chai A, Chung STL. Orientation information in encoding facial expressions. Vision research. 2018;150:29–37. 10.1016/j.visres.2018.07.001 . 30048659 PMC6139277

[16] Goffaux V, Duecker F, Hausfeld L, Schiltz C, Goebel R. Horizontal tuning for faces originates in high-level Fusiform Face Area. Neuropsychologia. 2016;81:1–11. 10.1016/j.neuropsychologia.2015.12.004 . 26683383

[17] Hashemi A, Pachai MV, Bennett PJ, Sekuler AB. The role of horizontal facial structure on the N170 and N250. Vision research. 2018 10.1016/j.visres.2018.02.006 . 29555299

[18] Jacques C, Schiltz C, Goffaux V. Face perception is tuned to horizontal orientation in the N170 time window. Journal of vision. 2014;14(2). 10.1167/14.2.5 . 24511144

[19] Pachai MV, Sekuler AB, Bennett PJ, Schyns PG, Ramon M. Personal familiarity enhances sensitivity to horizontal structure during processing of face identity. Journal of vision. 2017;17(6):5 10.1167/17.6.5 . 28593249

[20] Konar Y, Bennett PJ, Sekuler AB. Holistic processing is not correlated with face-identification accuracy. Psychological science. 2010;21(1):38–43. 10.1177/0956797609356508 . 20424020

[21] Keil MS. "I look in your eyes, honey": internal face features induce spatial frequency preference for human face processing. PLoS computational biology. 2009;5(3):e1000329 10.1371/journal.pcbi.1000329 . 19325870 PMC2653192

[22] Pachai MV, Bennett PJ, Sekuler AB. The effect of training with inverted faces on the selective use of horizontal structure. Vision research. 2018 10.1016/j.visres.2018.04.003 . 29678537

[23] Kalpadakis-Smith AV, Goffaux V, Greenwood JA. Crowding for faces is determined by visual (not holistic) similarity: Evidence from judgements of eye position. Scientific reports. 2018;8(1):12556 10.1038/s41598-018-30900-0 . 30135454 PMC6105622

[24] Goffaux V. The horizontal and vertical relations in upright faces are transmitted by different spatial frequency ranges. Acta psychologica. 2008;128(1):119–26. 10.1016/j.actpsy.2007.11.005 . 18166166

[25] Goffaux V, Rossion B. Face inversion disproportionately impairs the perception of vertical but not horizontal relations between features. Journal of experimental psychology Human perception and performance. 2007;33(4):995–1002. 10.1037/0096-1523.33.4.995 . 17683242

[26] Cabeza R, Kato T. Features are also important: contributions of featural and configural processing to face recognition. Psychological science. 2000;11(5):429–33. 10.1111/1467-9280.00283 . 11228917

[27] Schyns PG, Oliva A. Flexible, diagnosticity-driven, rather than fixed, perceptually determined scale selection in scene and face recognition. Perception. 1997;26(8):1027–38. 10.1068/p261027 . 9509161

[28] Gosselin F, Schyns PG. Bubbles: a technique to reveal the use of information in recognition tasks. Vision research. 2001;41(17):2261–71. . 11448718 10.1016/s0042-6989(01)00097-9

[29] Langton SR, Watt RJ, Bruce II. Do the eyes have it? Cues to the direction of social attention. Trends in cognitive sciences. 2000;4(2):50–9. . 10652522 10.1016/s1364-6613(99)01436-9

[30] Mareschal I, Calder AJ, Dadds MR, Clifford CW. Gaze categorization under uncertainty: psychophysics and modeling. Journal of vision. 2013;13(5):18 10.1167/13.5.18 . 23608340

[31] Vida MD, Maurer D. The development of fine-grained sensitivity to eye contact after 6 years of age. Journal of experimental child psychology. 2012;112(2):243–56. 10.1016/j.jecp.2012.02.002 . 22417921

[32] Gamer M, Hecht H. Are you looking at me? Measuring the cone of gaze. Journal of experimental psychology Human perception and performance. 2007;33(3):705–15. 10.1037/0096-1523.33.3.705 . 17563231

[33] Cheleski DJ, Mareschal I, Calder AJ, Clifford CW. Eye gaze is not coded by cardinal mechanisms alone. Proceedings Biological sciences. 2013;280(1764):20131049 10.1098/rspb.2013.1049 . 23782886 PMC3712425

[34] Stanislaw H, Todorov N. Calculation of signal detection theory measures. Behavior research methods, instruments, & computers: a journal of the Psychonomic Society, Inc. 1999;31(1):137–49. . 10495845 10.3758/bf03207704

[35] Hedges LV. Distribution theory for Glass’s estimator of effect size and related estimators. J Educ Behav Stat. 1981;6:107–28.

[36] Ando S. Perception of gaze direction based on luminance ratio. Perception. 2004;33(10):1173–84. 10.1068/p5297 . 15693663

[37] Ando S. Luminance-induced shift in the apparent direction of gaze. Perception. 2002;31(6):657–74. 10.1068/p3332 . 12092793

[38] Anstis SM, Mayhew JW, Morley T. The perception of where a face or television "portrait" is looking. Am J Psychol. 1969;82(4):474–89. . 5398220

[39] Calder AJ, Jenkins R, Cassel A, Clifford CW. Visual representation of eye gaze is coded by a nonopponent multichannel system. Journal of experimental psychology General. 2008;137(2):244–61. 10.1037/0096-3445.137.2.244 . 18473657

[40] Mareschal I, Calder AJ, Clifford CW. Humans have an expectation that gaze is directed toward them. Current biology: CB. 2013;23(8):717–21. 10.1016/j.cub.2013.03.030 . 23562265 PMC3918857

[41] Pellicano E, Rhodes G, Calder AJ. Reduced gaze aftereffects are related to difficulties categorising gaze direction in children with autism. Neuropsychologia. 2013;51(8):1504–9. 10.1016/j.neuropsychologia.2013.03.021 . 23583965 PMC3708125

[42] Taubert J, Goffaux V, Van Belle G, Vanduffel W, Vogels R. The impact of orientation filtering on face-selective neurons in monkey inferior temporal cortex. Scientific reports. 2016;6:21189 10.1038/srep21189 . 26879148 PMC4754760

[43] Mason MF, Hood BM, Macrae CN. Look into my eyes: gaze direction and person memory. Memory. 2004;12(5):637–43. 10.1080/09658210344000152 . 15615320

[44] Macrae CN, Hood BM, Milne AB, Rowe AC, Mason MF. Are you looking at me? Eye gaze and person perception. Psychological science. 2002;13(5):460–4. 10.1111/1467-9280.00481 . 12219814

[45] Smith AD, Hood BM, Hector K. Eye remember you two: gaze direction modulates face recognition in a developmental study. Developmental science. 2006;9(5):465–72. 10.1111/j.1467-7687.2006.00513.x . 16911448

[46] Nummenmaa L, Calder AJ. Neural mechanisms of social attention. Trends in cognitive sciences. 2009;13(3):135–43. 10.1016/j.tics.2008.12.006 . 19223221

