# Supplementary material for: Fixed or flexible? Orientation preference in identity and gaze processing in humans
Source: PLoS One. 2019 Jan 25;14(1):e0210503. doi: 10.1371/journal.pone.0210503 (PMC6347268; doi:10.1371/journal.pone.0210503)
Supplement: S1 Fig — A. Illustration of the congruent-same (i.e., fully identical faces) and different-incongruent (i.e., identity differences restricted to the eye region) pairs of the congruency paradigm (adapted from Fig 7 in [8]). B. Group-averaged sensitivity to identity differences restricted to the eye region. Error bars are 95% confidence intervals. (DOCX) [file pone.0210503.s001.docx]

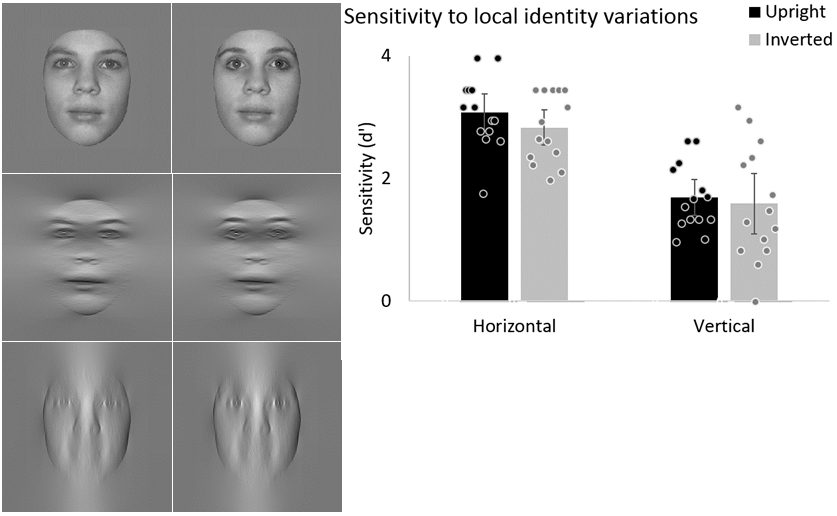


**S1 Fig. Sensitivity to local identity variations.** **A.** Illustration of the congruent-same (i.e., fully identical faces) and different-incongruent (i.e., identity differences restricted to the eye region) pairs of the congruency paradigm (adapted from Goffaux and Dakin, 2010; Figure 7). **B.** Group-averaged sensitivity to identity differences restricted to the eye region. Error bars are 95% confidence intervals.
