# Supplementary material for: Fixed or flexible? Orientation preference in identity and gaze processing in humans
Source: PLoS One. 2019 Jan 25;14(1):e0210503. doi: 10.1371/journal.pone.0210503 (PMC6347268; doi:10.1371/journal.pone.0210503)
Supplement: S2 Fig — Since the focus of this work is on the orientation dependence of identity and gaze processing, image analyses were performed but then averaged across spatial frequencies. Here the plot shows the average image energy differences resulting from variations in gaze directions (as plotted on Fig 1a, right plot) as a function of orientation selectively in the low, middle, and high SF bands of the eye image. This plots indicates that the vertical predominance of gaze direction cues is most notable in the low to mid SF. At high SF, gaze shifts the horizontal fine structure of the eye likely due to the slight displacement of the lower lid (see Fig 1b). Error bars are 95% confidence intervals. (see S1 File). (DOCX) [file pone.0210503.s002.docx]

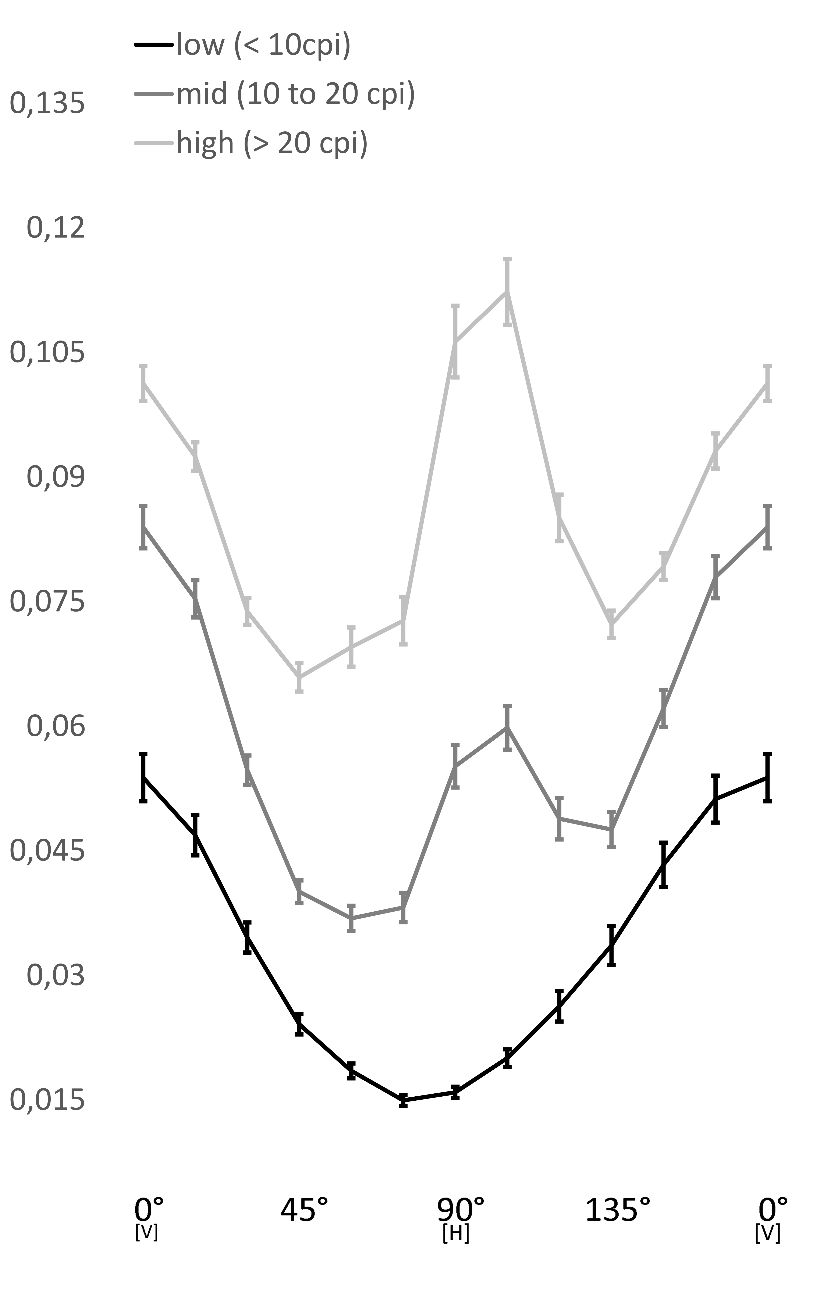


**S2 Fig. Orientation profile of gaze direction variations in low, medium and high spatial frequencies.** Since the focus of this work is on the orientation dependence of identity and gaze processing, image analyses were performed but then averaged across spatial frequencies. Here the plot shows the average image energy differences resulting from variations in gaze directions (as plotted on Figure 1a, right plot) as a function of orientation selectively in the low, middle, and high SF bands of the eye image. This plots indicates that the vertical predominance of gaze direction cues is most notable in the low to mid SF. At high SF, gaze shifts the horizontal fine structure of the eye likely due to the slight displacement of the lower lid (see Figure 1b). Error bars are 95% confidence intervals. (see Methods, Image analyses for a full description of the image analyses).
