# Supplementary material for: Fixed or flexible? Orientation preference in identity and gaze processing in humans
Source: PLoS One. 2019 Jan 25;14(1):e0210503. doi: 10.1371/journal.pone.0210503 (PMC6347268; doi:10.1371/journal.pone.0210503)
Supplement: S1 File — We evaluated how stimulus variations in gaze direction and identity influenced image energy profile across orientations. For gaze direction, we delineated the eye region in the 11 full-spectrum images (one for each gaze direction), replaced all pixels outside this region by grey values, and cropped the image to fit eye region borders in order to capture energy variations selectively induced by gaze shifts. For identity, we analysed the egg-shaped 20 face identities used in the face identification experiment. (DOCX) [file pone.0210503.s003.docx]

**S1 Dataset. Image analyses.**

We evaluated how stimulus variations in gaze direction and identity influenced image energy profile across orientations. For gaze direction, we delineated the eye region in the 11 full-spectrum images (one for each gaze direction), replaced all pixels outside this region by grey values, and cropped the image to fit eye region borders in order to capture energy variations selectively induced by gaze shifts. For identity, we analysed the egg-shaped 20 face identities used in the face identification experiment.

We followed the same procedure as in Kalpadakis-Smith et al. [1]. To avoid border effects, images were padded to extend the image dimensions by 160x160 pixels beyond the borders of the eye region or the egg-shaped aperture. Next their mean luminance and RMS contrast were set to 0 and 1, respectively. The amplitude and phase spectrum for each image were then obtained by means of a two-dimensional fast Fourier Transform and multiplied with wrapped Gaussian filters, with peak orientations centred on values from 0° to 165° in thirteen 15° steps (15° standard deviation) and peak spatial frequencies sampled in twenty linear steps between 2-60 cycles per image (one octave bandwidth). Filtered images were inverse-Fourier-transformed. Energy of the so-filtered images was summed across SF and squared. We obtained one mean energy value per orientation by averaging across gaze directions and identities. Note that because the Fourier transform represents image energy in a discrete manner, energy at the lowest spatial frequency components can only be reliably sampled at the main cardinal and oblique ranges (i.e., 0°, 45°, 90°, 135°) especially when very narrow orientation filters are used [2]. However because we made use of relatively large orientation filters and we were mainly interested by energy measures at cardinal orientations, this issue does not influence our conclusions. As can be seen on Figure 1a, both the whole face image and the eye region contained most of their energy in the H range.

We next sought to characterize energy differences resulting from variations in gaze direction and identity. We compared the distinct identities and gaze directions two-by-two by image subtraction (i.e., on a pixel-by-pixel basis) in each spatial frequency and orientation band separately. Figure 1b maps the normalized average energy differences related to identity and gaze variations in image space. Next, in each orientation range, differences were summed across SF and image, squared, and then normalized by total energy. Last, we averaged this total energy difference across (identity and gaze) comparisons. We found variations in identity to be distributed across a large range of orientations; however differences at the level of the main facial features, i.e. the most diagnostic cues to identity, were prominently conveyed in the H range (Figure 1b). In contrast, lateral gaze shifts were primarily conveyed by V range, with the other ranges signalling the more subtle displacements of the lid across shifts. To more directly visualize the relative contribution of each cardinal orientation, we divided the total energy difference in the H range from the energy difference in the V range; values above 1 indicate that differences in energy are largest in H whereas values below 1 indicate largest differences in the V range. As the inset of Figure 1a illustrates, gaze direction shifts manifested themselves through substantial variations in vertically-oriented energy. In contrast, identity-related energy variations were only slightly larger in the H than the V range.

**References**

1. Kalpadakis-Smith AV, Goffaux V, Greenwood JA. Crowding for faces is determined by visual (not holistic) similarity: Evidence from judgements of eye position. Scientific reports. 2018;8(1):12556. doi: 10.1038/s41598-018-30900-0. PubMed PMID: 30135454; PubMed Central PMCID: PMCPMC6105622.

2. Hansen BC, Essock EA. A horizontal bias in human visual processing of orientation and its correspondence to the structural components of natural scenes. Journal of vision. 2004;4(12):1044-60. doi: 10.1167/4.12.5. PubMed PMID: 15669910.
