# Supplementary material for: Fixed or flexible? Orientation preference in identity and gaze processing in humans
Source: PLoS One. 2019 Jan 25;14(1):e0210503. doi: 10.1371/journal.pone.0210503 (PMC6347268; doi:10.1371/journal.pone.0210503)
Supplement: S2 File — A notable difference between the identity and the gaze experiments described in the main manuscript is the spatial extent of the task-relevant cues. Gaze cues are by definition local and participants were instructed to focus on the eyes to categorize gaze direction. In contrast, in the identity experiment, participants had to match whole faces and identity variations extended to the whole face surface. To address whether face identity still best processed in the horizontal range when based on local identity cues, we re-analysed the behavioral data of the experiment 4 reported by Goffaux and Dakin (2010), in which participants were instructed to process faces locally. (DOCX) [file pone.0210503.s004.docx]

**S2 Dataset. Sensitivity to local identity variations.**

A notable difference between the identity and the gaze experiments described in the main manuscript is the spatial extent of the task-relevant cues. Gaze cues are by definition local and participants were instructed to focus on the eyes to categorize gaze direction. In contrast, in the identity experiment, participants had to match whole faces and identity variations extended to the whole face surface. Is this difference in the spatial extent of task-relevant information contributing to our findings? In other words, is face identity still best processed in the horizontal range when based on local identity cues?

To address this question, we re-analysed the behavioral data of the experiment 4 reported by Goffaux and Dakin (2010), in which participants were instructed to process faces locally. In this study, we used a so-called congruency paradigm; participants performed a same/different matching task on the eye region (e.g., eyes and brows) of whole face stimuli, while ignoring distractor features (e.g., nose and mouth). We manipulated the congruency of target and distractor features (Goffaux and Dakin, 2010; Figure 7A). In congruent conditions, both the target and distractor features are same or different, therefore leading to an identical response. In incongruent conditions, target and distractor features call for opposite responses (be that same or different). Thus, there were four crossed conditions (same-congruent, different-congruent, same-incongruent, and different-incongruent) tested using upright and inverted displays under two filter-orientation conditions (horizontal and vertical). The present analyses focus on the different-incongruent conditions where cues to identity were strictly local and confined to the eye region like in the main gaze experiment.

The orientation-filtering method was identical to the main experiments (20°-standard deviation orientation filters). For a full description of the methodology, please see Goffaux and Dakin (2010). We estimated individual sensitivity (d′) based on hits in the different-incongruent condition and correct rejections in the congruent-same condition. Sensitivity measures were submitted to a 2 × 2 repeated-measure ANOVA with Orientation content (H, V) and Planar Orientation (Upright, inverted) as within-subject factors.

Only the main effect of Orientation content was significant (F(1,13) = 64.94, p< .000001, partial η²= .83). The processing of local eye identity cues was indeed best based on the horizontal content of the face image. Neither the main effect of Planar Orientation, nor the interaction between these factors were significant (F(1, 13)= 2.73, p= .12, partial η²= .17 and F(1, 13)= .23, p= .64, partial η²= .017). The absence of inversion effect replicates past evidence that inversion often spares the local processing of feature differences across identities [1, 2].

To sum up, faces are best identified based on horizontal cues even when human observers are instructed to focus on local cues to identity. Future studies should investigate the respective contribution of the eyes and brows to this pattern of behavioral performance. Findings by Duncan and colleagues ([3]) are informative with this respect. These authors found that the preferential reliance on horizontal information when processing facial expression is functionally linked to the utilization on the information contained in the eyes and not brows of face stimuli. In other words, it is likely that the processing of the eyes contributed largely to the horizontal preference observed in the different-incongruent condition of the congruency experiment.

**References**

1. McKone E, Yovel G. Why does picture-plane inversion sometimes dissociate perception of features and spacing in faces, and sometimes not? Toward a new theory of holistic processing. Psychonomic bulletin & review. 2009;16(5):778-97. doi: 10.3758/PBR.16.5.778. PubMed PMID: 19815781.

2. Goffaux V. The discriminability of local cues determines the strength of holistic face processing. Vision research. 2012;64:17-22. doi: 10.1016/j.visres.2012.04.022. PubMed PMID: 22613193.

3. Duncan J, Gosselin F, Cobarro C, Dugas G, Blais C, Fiset D. Orientations for the successful categorization of facial expressions and their link with facial features. Journal of vision. 2017;17(14):7. doi: 10.1167/17.14.7. PubMed PMID: 29228140.
